# Fine tuning of virulence regulatory pathways in enteric bacteria in response to varying bile and oxygen concentrations in the gastrointestinal tract

**DOI:** 10.1186/s13099-014-0038-9

**Published:** 2014-09-09

**Authors:** Chirantana Sengupta, Sreejana Ray, Rukhsana Chowdhury

**Affiliations:** 1 Infectious Diseases and Immunology Division, CSIR-Indian Institute of Chemical Biology, 4 Raja S. C. Mullick Road, Kolkata 700032, India; 2 Academy for Scientific and Innovative Research, CSIR-IICB Campus, Kolkata 700032, India

**Keywords:** V. cholerae, Shigella, Salmonella, Virulence, Bile, Oxygen concentration

## Abstract

After entering the gastrointestinal (GI) tract on the way to their physiological site of infection, enteric bacteria encounter a remarkable diversity in environmental conditions. There are gross differences in the physico-chemical parameters in different sections of the GI tract e.g. between the stomach, small intestine and large intestine. Furthermore, even within a certain anatomical site, there are subtle differences in the microenvironment e.g. between the lumen, mucous layer and epithelial surface. Enteric pathogens must not only survive passage through the rapidly changing environments encountered at different niches of the GI tract but must also appropriately coordinate expression of virulence determinants in response to environmental cues at different stages of infection. There are some common themes in the responses of enteric pathogens to environmental cues, there are also distinct differences that may reflect differences in basic pathogenesis mechanisms. The role of bile and oxygen concentration in spatiotemporal regulation of virulence genes in selected enteric pathogens has been reviewed.

## 1 Introduction

Enteric and diarrheal diseases remain a major cause of human mortality and morbidity, particularly in developing countries where poor sanitation, lack of safe drinking water and other factors contribute to regular infections specially in children and the aged population. It is estimated that enteric infections are a major cause of childhood mortality in developing countries, killing nearly 1 million children under age 5 every year (WHO report 2013). Repeated episodes of severe diarrhea in the early years of life cause malnutrition and impaired cognitive development which have serious lifelong implications. Some of the most common enteric diseases are caused by bacterial pathogens like *Vibrio cholerae, Salmonella enterica, Shigella flexneri*, *Escherichia coli* etc. Development of resistance to multiple antimicrobial drugs among these pathogens is increasing alarmingly with serious consequences on public health (WHO report 2014).

Pathogenic bacteria generally produce an array of virulence factors that allow them to overcome host defenses and survive and multiply in the host body. Disease is often incidental to this primary process. Thus virulence factors include toxins, factors required for adherence to and invasion of host cells, factors conferring resistance to host defenses, those required for modulation of hostile host environments into more favourable ones, and a plethora of accessory factors. Expression of the virulence genes is often tightly regulated by environmental, metabolic and other factors. Many bacterial pathogens have evolved to recognize specific parameters characteristic of the human body as cues for production of virulence factors. The intestinal environment provides a variety of physico-chemical cues to enteric bacteria [1]–[5]. Bile is a major constituent of the small intestine secreted into the lumen of the duodenum through the bile duct and is thus likely to be encountered by intestinal bacteria in the early stages of infection. Bile is sensed as a host specific cue and regulates expression of virulence factors in certain bacterial pathogens [6]. Also oxygen concentration, presumed to be low in the intestine, is sensed by bacteria and there is some evidence that enteric pathogens express virulence factors in response to low oxygen concentrations [7],[8]. Furthermore, since concentration of bile or oxygen differs in different anatomical sites of the intestine, localized microenvironments may impose spatio-temporal controls to fine tune virulence gene expression at different stages of infection. The virulence regulatory systems of a few enteric bacteria and their fine tuning by bile and oxygen concentration is reviewed.

### 1.1 Virulence regulatory pathways

**Figure 1:**
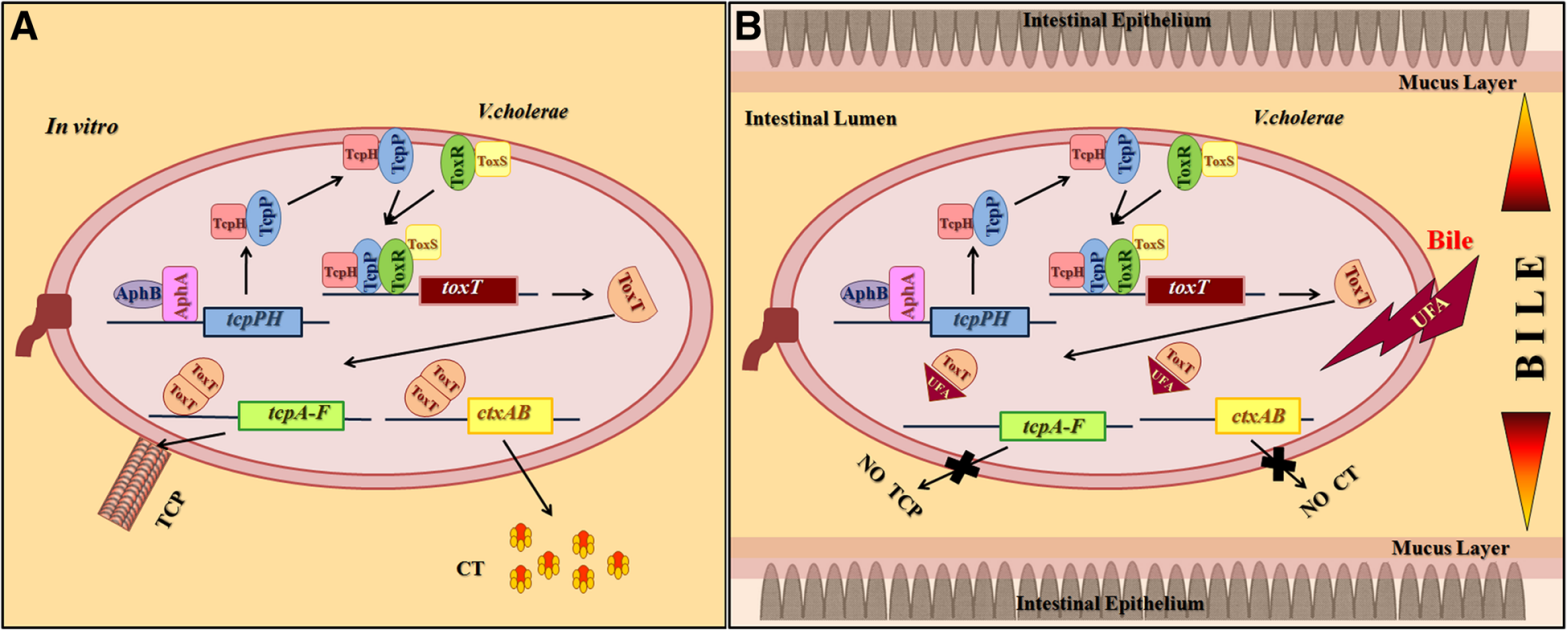
**Effect of bile on*V. cholerae*virulence regulation. A**. When *V. cholerae* is grown *in vitro* under permissive conditions without bile the ToxR regulon is activated and the major virulence factors CT and TCP are produced. **B**. In the intestinal lumen where bile concentration is high, UFA present in bile prevents dimerization of ToxT and thus expression of the virulence genes *ctxAB* and *tcpA* is reduced. As the bacteria move away from the lumen through the mucus layer to the underlying epithelium, concentration of bile gradually decreases, consequently *ctxAB* and *tcpA* expression increases.

Pathogenic bacteria produce an array of virulence factors that allow them to overcome host defenses and survive and multiply in the host body. In *V. cholerae*, the major virulence factors are cholera toxin (CT) and the colonization factor, toxin coregulated pilus (TCP). Expression of the corresponding genes, *ctxAB* and *tcpA* is coordinately regulated by transcriptional regulators of the ToxR regulon [9],[10]. At the top of the hierarchy are AphA, a winged-helix transcription factor, and AphB, a LysR-type regulator, that together activate expression of the *tcpPH* genes. TcpP/TcpH and another transcription regulator ToxR, act synergistically to promote transcription of the *toxT* gene. ToxT dimers, in turn, activate expression of several virulence genes, including *ctxAB and tcpA* (Figure 1A) [11]–[16]. Although the *V. cholerae ctxAB* is closely related to the *eltAB* genes of *E. coli*, the latter is not part of a regulatory cascade and is known to be regulated by cAMP-CRP and the histone-like nucleoid structuring protein H-NS under glucose limiting conditions and low temperature respectively [17],[18]. Both cAMP-CRP and H-NS also affect *ctxAB* expression but by different mechanisms as compared to their effect on *eltAB* expression. Whereas cAMP-CRP binds to three sites upstream of the *E. coli eltAB* gene preventing open complex formation and thereby repressing transcription [18], in *V. cholerae* cAMP-CRP represses AphA/AphB-dependent transcriptional activation of the master regulator *tcpPH* as a result of which, due to a cascading effect, *ctxAB* expression is reduced [19]. In *V. cholerae* the global silencer H-NS binds with high affinity to the *ctxAB* promoter repressing transcription, the transcription activator ToxT functions as an anti-repressor and is required to overcome the H-NS mediated repression [20]. In contrast, in *E. coli*, H-NS represses *eltAB* expression by binding downstream of the transcription start site and aborting transcription elongation at low temperatures, at higher temperatures the repression is partially relieved probably due to changes in DNA curvature that reduce the affinity of H-NS for its binding sites [17]. A similar repression of virulence genes by H-NS at low temperatures that is relieved at 37°C has also been reported in *S. flexneri*[21]. Expression of the *S. flexneri* virulence regulon is controlled by a regulatory cascade in which the master regulator VirF activates expression of *virB* and the structural gene *icsA*. VirB in turn activates transcription of several virulence genes including those coding for invasion proteins and a type III secretion system (T3SS). The transcriptional regulation of *virF* is complex and is controlled by the global regulators H-NS, Fis, and IHF, as well as the pH-responsive regulator CpxR [21]–[24]. Although mechanistically different, VirB is functionally similar to the *V. cholerae* ToxT since both function as antirepressors to overcome H-NS mediated gene silencing [25].

Two distinct pathogenecity islands SPI-1 and SPI-2 in the *Salmonella* chromosome encode two distinct virulence associated T3SS, the components of which are expressed in a tightly regulated hierarchical manner at different stages of infection. SPI-1 encoded T3SS is activated by contact with host epithelial cells and translocates effector proteins across the plasma membrane. After the bacteria invade the epithelial cells, the SPI-2 T3SS is expressed within the phagosome and translocates effectors across the vacuolar membrane. At the top of the SPI-1 virulence regulatory cascade are the AraC family transcription factors, HilC and HilD that together with RtsA, encoded in the core genome, can regulate their own as well as each other’s expression through a complex feedback and feedforward mechanism. Each of these regulators can also activate expression of the master regulator HilA that in turn activates expression of the genes encoding the structural components of the SPI-1 T3SS. Unlike SPI-1, SPI-2 encodes a single cognate regulator, the sensor kinase SsrA and the response regulator SsrB [26]–[28]. Crosstalk between SPI-1 and SPI-2 through HilD induces expression of *ssrAB*[29]*.* These core regulatory circuits are modulated by a number of global regulators like H-NS, FIS, OmpR, PhoPQ etc. [30]–[32].

### 1.2 Recognizing and responding to environmental signals

A feature of the adaptation strategy of pathogens for optimal survival in their hosts is the recognition of physico-chemical parameters characteristic of the host body and expression of genes related to survival and virulence in response to cues specific to localized microenvironments within the host. This may be achieved either by direct recognition of environmental parameters by dedicated proteins or by integration of virulence gene expression with regulatory circuits that respond to environmental conditions usually involving specific adaptor molecules (1, 2, 4, 5). The regulation of virulence gene expression by bile and oxygen concentration characteristic of the intestinal lumen and their spatiotemporal control will be discussed.

### 1.3 Bile mediated virulence regulation

Bile is secreted into the lumen of the duodenum from the gall bladder through the bile duct and is inevitably encountered by all enteric bacteria in their human hosts. Bile salts have detergent-like activity [8] that can cause disaggregation of the lipid bilayer structure of cellular membranes, a property that is necessary for protection of the host from invading bacteria. However, Gram-negative enteric bacteria are inherently resistant to bile, partly due to the basic, asymmetric structure of their outer membranes (OM). The protection provided by the OM is partial and the OM must function in conjunction with energy-dependent efflux systems, which can transport vectorially a diverse array of compounds with little chemical similarity [33]. The best characterized is the AcrAB-TolC efflux pump that can exclude substrates with diverse specificity from the bacteria and thus confers intrinsic resistance to a wide variety of compounds. The AcrAB-TolC efflux pump is required for growth of a number of enteric bacteria including *E. coli*, *V. cholerae* and salmonellae, in the presence of bile [34]–[36]. In addition, salmonellae that has to survive under very high concentrations of bile in the gall bladder employ a number a strategies including peptidoglycan remodeling and changes in outer membrane porins to adapt to high bile concentrations. Furthermore, high level of resistance to bile requires the pleotropic virulence regulator PhoPQ although no individual PhoP-activated (*pag*) or -repressed (*prg*) gene could be identified that might have a role in bile resistance [37].

Interestingly, many enteric bacteria have further adapted to recognize bile as a signal indicating their entry into the human host, to which they respond by inducing the production of virulence and other factors necessary for survival in the host. Several studies have revealed pathogen specific responses to bile that alter the expression of virulence factors. When *V. cholerae* was grown in the presence of bile, expression of the essential virulence genes *ctxAB* and *tcpA* was drastically repressed [38]. Since bile is a heterogeneous mixture, crude bile was fractionated, and the components that mediate virulence gene repression were identified. It was shown that the unsaturated fatty acids (UFAs) present in bile, arachidonic, linoleic, and oleic acids were responsible for repression of *ctxAB* and *tcpA* genes [39]. However, expression of the transcriptional activator *toxT* was not affected and there was no significant difference in ToxT protein levels between *V. cholerae* grown in the presence or absence of bile or UFAs [39]. Subsequently, the crystal structure of ToxT revealed that UFAs can bind to ToxT and keep ToxT in a ‘closed’ conformation that is not capable of binding DNA [40]. Hence ToxT cannot activate expression of *ctxAB* and *tcpA* in the presence of bile or UFAs and the genes continue to be repressed by H-NS [41]. Bile also causes drastic repression of virulence genes in salmonellae. Salmonellae grown in the presence of bile demonstrated a marked repression of invasion genes and consequently the ability to invade epithelial cells was significantly reduced. Transcription assays demonstrated that bile exerted its effect at the top of the invasion regulatory cascade involving SirA and BarA [42].

A common theme in *V. cholerae* and salmonellae virulence regulation appears to be repression of essential virulence factors in the presence of bile. It is paradoxical that bile, that would inevitably be encountered by all enteric bacteria early in infection should repress expression of factors required for virulence in two important and successful enteric pathogens. It is attractive to hypothesize that these pathogens have co-opted bile as an environmental cue to determine their location with respect to different anatomical sites within the intestine so that a spatio-temporal pattern of virulence factor expression could be established. It is known that the concentration of bile gradually decreases from the intestinal lumen to the epithelial surface. In the intestinal lumen where bile concentration is high, CT and TCP production would be shut off in infecting *V. cholerae*. It might be beneficial for *V. cholerae* to shut off production of CT and TCP in the intestinal lumen for the following reasons. TCP, a pilus if produced in the intestinal lumen might impede progress of the bacteria through the mucus lining of the intestine and retard passage to the underlying epithelial cells. CT production early in infection might lead to profuse fluid secretion from the epithelial cells that could wash away bacteria in the lumen before they could adhere to the epithelial layer. Of greater importance in the early stages of infection would be motility and chemotaxis that would enable the infecting *V. cholerae* to penetrate the mucus layer and adhere to the underlying epithelial cells. Indeed, bile upregulates expression of flagellar genes and motility in *V. cholerae*[38]. Once the bacteria reach the epithelial layer, the lower bile concentration in this microenvironment would allow the repression of virulence genes to be overcome and CT and TCP would be produced (Figure 1B). Similarly, in *S*. Typhimurium, a model has been proposed where bile is recognized as an environmental signal to repress its invasive capacity in the lumen of the intestine, after penetration of the mucous layer and attachment to intestinal epithelial cells, since bile concentration gradually decreases, invasion genes would be expressed and invasion would be initiated. However, although it would be reasonable to assume that bacterial motility would be required to pass through the lumen, and across the mucous membrane to reach the epithelial cells, transcriptome analysis has indicated a bile dependent reduction in expression of motility genes in *S.* Typhimurium, the reason for which is not clear.

The effect of bile on the invasion phenotype of *S. flexneri* is very different from that in the closely related salmonellae. Bile induces production of two outer membrane proteins OspE1/OspE2 that function to promote initial adherence of *S. flexneri* to the intestinal epithelium [43]. Furthermore, when *S. flexneri* was grown in the presence of deoxycholate (DOC), it was capable of enhanced adherence to and invasion of epithelial cells as compared to bacteria grown without DOC. It has been demonstrated that DOC binds to the invasion protein IpaD at the tip of the external needle of the T3SS to induce recruitment of the translocator protein IpaB into the maturing tip complex thereby facilitating invasion of epithelial cells [44].

### 1.4 Oxygen concentration dependent regulation of virulence

Oxygen concentration in the intestine is generally considered to be low and anaerobiosis has been shown to regulate virulence of some pathogens. Bacterial adaptation to growth under oxygen deprivation involves several global regulatory systems, like FNR and ArcA/ArcB [45],[46]. FNR functions as a regulator of virulence genes in several pathogens. FNR positively regulates the expression of several loci involved in flagellar biosynthesis, chemotaxis, acetate metabolism, and SPI-1 invasion genes that have a significant role in *Salmonella* pathogenesis [47]. FNR also has profound effects on virulence gene expression in *S. flexneri*, although in *V. cholerae* it has been reported that FNR has no role in virulence regulation [46],[48].

**Figure 2:**
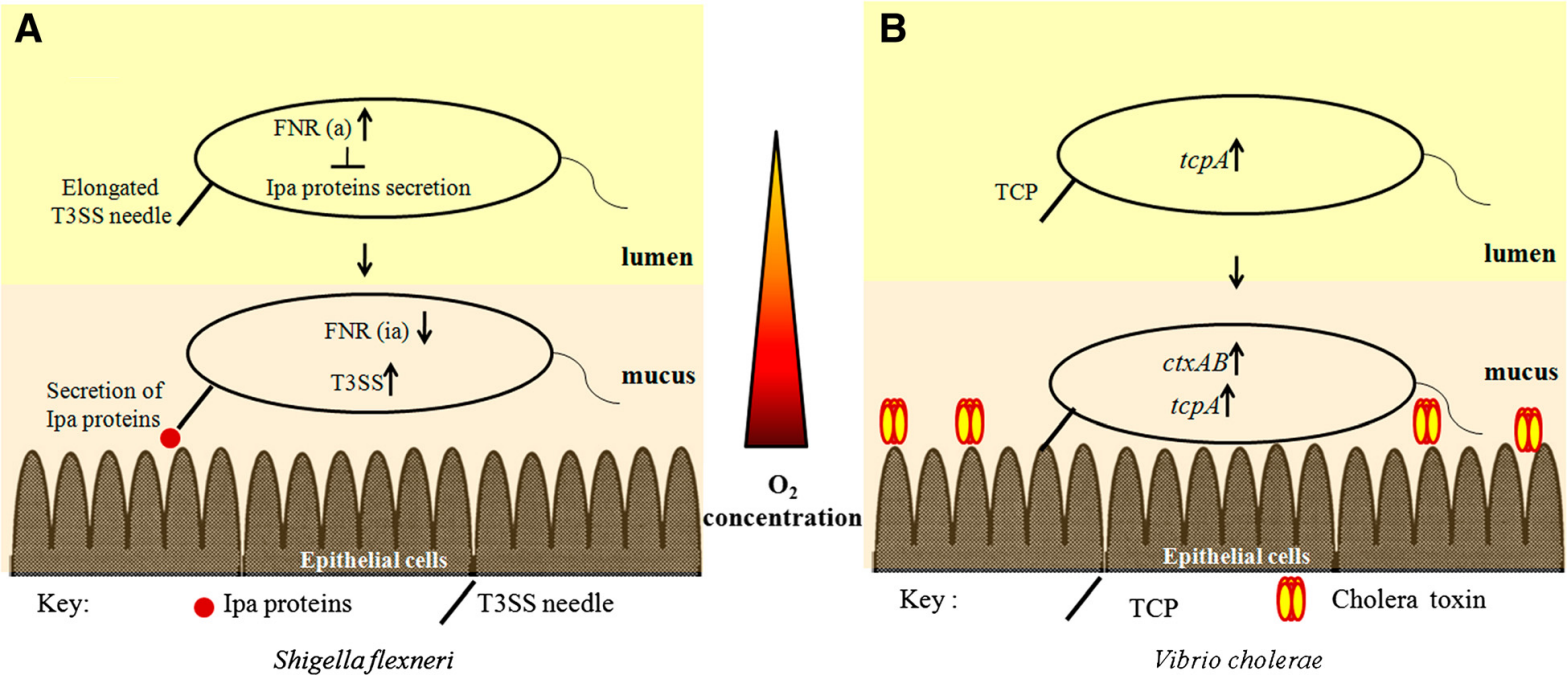
**Spatio-temporal regulation of virulence by oxygen*.*** The concentration of oxygen is low in the intestinal lumen but increases adjacent to the mucosal surface. **A**. In *S. flexneri* microaerobic condition of the lumen activates FNR [FNR(a)] which in turn represses Ipa secretion. Extended T3SS needles are formed. As the bacteria moves to the zone of higher oxygen concentration adjacent to the epithelial layer, FNR is inactivated [FNR(ia)] and T3SS effector genes are induced. **B**. In *V. cholerae,* the *tcpA* gene, but not *ctxAB*, is induced under the microaerobic condition of the lumen, when the bacteria reach the epithelial layer, the relatively higher oxygen concentration allows expression of both *tcpA* and *ctxAB*.

Although low oxygen concentration is certainly a characteristic feature of the intestinal lumen, it has been elegantly demonstrated that there is a zone of relative oxygenation adjacent to the GI tract mucosa probably due to diffusion from the capillary network at the tips of the villi. Varying oxygen concentrations at different anatomical sites of the GI tract govern activity of the T3SS of *S. flexneri* and consequently cell invasion and virulence. Under the microaerobic condition of the intestinal lumen, the anaerobic response regulator FNR is activated and *S. flexneri* produces extended T3SS needles but secretion of Invasion plasmid antigen (Ipa) effector molecules is reduced in a FNR dependent manner. However, after reaching the relatively aerobic zone adjacent to the mucosal surface, FNR is inactivated and T3SS activation occurs, consequently effectors are secreted (Figure 2). This finely tuned strategy primes the bacteria for invasion while they are in the intestinal lumen but T3SS activation occurs only at its precise site of action, adjacent to the epithelial layer, enhancing invasion and virulence [48].

Spatiotemporal control of virulence gene expression was also observed in the infant mouse model of cholera following *V. cholerae* infection. Using the RIVET technology, it has been demonstrated that expression of the colonization factor TCP encoded by the t*cpA* gene was induced biphasically in two temporally and spatially separable events, whereas the *ctxAB* gene encoding CT was induced monophasically concomitant with the second phase of *tcpA* induction (Figure 2) [49]. In separate studies it has been demonstrated that under anaerobic conditions *tcpA* is expressed but *ctxAB* is repressed by H-NS. Thus, although expression of *ctxAB* and *tcpA* is coordinately regulated *in vitro*, the coordinate regulation is lost *in vivo* and also under anaerobic conditions [50]. Therefore it is reasonable to hypothesize that the microaerobic condition of the intestinal lumen might allow TcpA production, priming the bacteria for colonization preceding production of CT that is initiated in a later stage of infection, probably when bacteria reaches the epithelial layer where aeration is relatively high.

### 1.5 Conclusions and future directions

Many studies have demonstrated that enteric pathogens have evolved to recognize and respond to gross as well as subtle changes in environmental parameters that characterize different niches of the GI tract. The effect of a variety of physico-chemical parameters on expression of virulence genes have been studied in *in vitro* and *in vivo* systems, and fine tuning of gene expression at different stages of infection has been established. However, the bacterial sensors of environmental cues are less well characterized. Also few studies have been directed towards identifying changes in the membrane of pathogens at different stages of infection, which could be important in the context of designing vaccines. Finally, although the effect of a variety of physico-chemical parameters and also some metabolites have been studied, the effect of the resident microbiota on gene expression of invading pathogens is not yet understood and could be a focus of future studies.

## Competing interests

The authors declare that they have no competing interests.

## Authors’ contribution

CS, SR and RC collected information and drafted the manuscript. RC wrote the final manuscript. All authors read and approved the final manuscript.
